# Photoluminescence color-tuning with polymer-dispersed fluorescent films containing two fluorinated diphenylacetylene-type fluorophores

**DOI:** 10.3762/bjoc.20.225

**Published:** 2024-10-23

**Authors:** Kazuki Kobayashi, Shigeyuki Yamada, Motohiro Yasui, Tsutomu Konno

**Affiliations:** 1 Faculty of Molecular Chemistry and Engineering, Kyoto Institute of Technology, Matsugasaki, Sakyo-ku, Kyoto 606-8585, Japan

**Keywords:** energy transfer, fluorinated diphenylacetylenes, photoluminescence, polymer-dispersed films, white luminescence

## Abstract

The development of organic light-emitting devices has driven demand for new luminescent materials, particularly after the 2001 discovery of aggregation-induced emission. This study focuses on fluorinated diphenylacetylene-based luminescent molecules, revealing that specific molecular modifications can enhance fluorescence and achieve a wide range of photoluminescence colors. A simple and effective luminescence color-tuning method is proposed to investigate the photoluminescence behavior of two-component polymer dispersion films blended with two types of fluorinated diphenylacetylenes, namely blue- and yellow- or red-fluorescent fluorinated diphenylacetylenes. It is confirmed that if blue and green–yellow or yellow fluorophores are blended in appropriate ratios, a binary blend with color coordinates (0.20, 0.32) can be achieved, which approaches the white point of pure white emission. These findings contribute to the development of effective lighting and display devices as new white-light-emitting materials.

## Introduction

Luminescent materials in lighting and display devices have become indispensable in daily life [1–3]. In recent years, organic electroluminescent devices have attracted significant attention as lightweight and energy-saving optical devices, and there has been a strong demand for the development of luminescent materials. Until now, the design of solid-state light-emitting materials has not been established, and therefore, their development has been severely delayed [4–6]. However, since Tang et al. first reported the aggregation-induced emission phenomenon in 2001 [7], the development of solid-state light-emitting materials has accelerated significantly [8–10].

**Figure 1:**
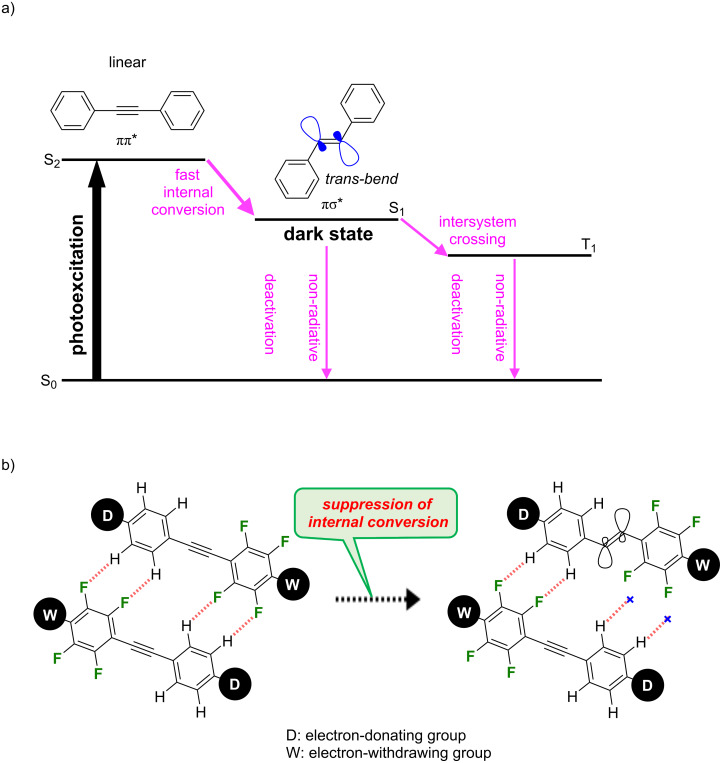
(a) Reported photophysics of diphenylacetylene after photoexcitation. (b) Our molecular design to suppress internal conversion.

Many photoluminescent materials that emit blue, green, and yellow photoluminescence (PL) have been developed, whereas red PL with PL wavelengths in the long wavelength region is considered difficult to achieve owing to the energy gap law [11–13]. Over the past few decades, our group has been vigorously pursuing the exploration of functional molecules with a linear diphenylacetylene structure as the π-conjugated core. As a part of our research projects, we have begun to explore diphenylacetylene-based luminescent molecules despite diphenylacetylene not exhibiting fluorescence at room temperature because it undergoes a πσ* excited state that rapidly forms a *trans*-bend structure (Figure 1a) [14–16].

**Figure 2:**
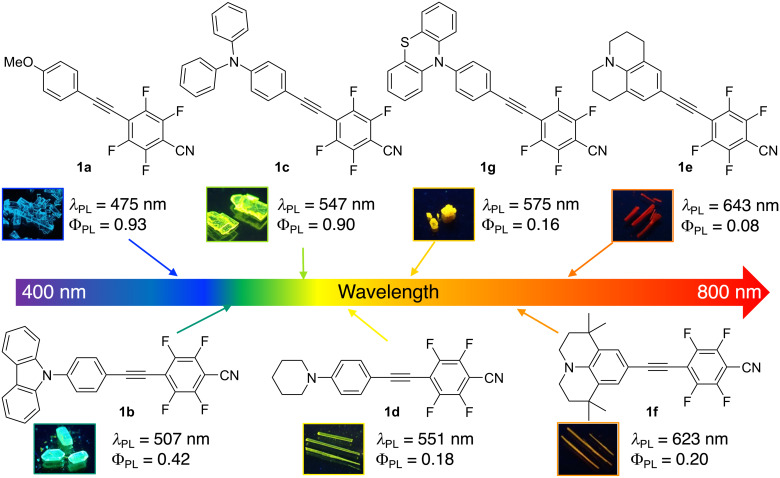
Relationship between the molecular structure of fluorinated diphenylacetylenes and photoluminescence (PL) color in their crystalline states.

Our extensive efforts have revealed that introducing electron-donating alkoxy and electron-withdrawing cyano groups at both ends of the diphenylacetylene scaffold slows the internal conversion to the πσ* excited state. Further incorporating four fluoro substituents in the short-axis direction of the electron-deficient aromatic ring significantly retards the internal conversion via the formation of H···F hydrogen bonds, leading to a marked blue fluorescence in the crystalline state (Figure 1b) [17–20]. Recently, the introduction of *N,N*-disubstituted amino groups as electron-donating groups was shown to promote intramolecular charge transfer (ICT) and shifted the PL wavelength to longer wavelengths, resulting in yellow or orange fluorescence in the solid state [21–22]. In addition, cross-linking between the amino group and attached benzene ring effectively suppresses the formation of the twisted ICT state, resulting in red fluorescence even in the solid state (Figure 2) [23].

The precise tuning of molecular and electronic structures has made it possible to produce a wide range of PL colors. However, the development of white-light-emitting materials, which are especially indispensable for our affluent life, is more difficult than the development of the blue-, yellow-, and red-light-emitting molecules mentioned above [24–26]. Therefore, to achieve white luminescence covering the entire spectral range of the visible light region, two or more colors of fluorescence or phosphorescence from different luminescent centers in the polymer matrix should be combined, and the PL color can be precisely tuned by controlling the ratio of the PL luminescent materials [27–29]. In this study, we prepared polymer dispersion fluorescent films containing two compounds from our fluorinated diphenylacetylene library that exhibit different PL characteristics from blue to red in the solid state, as shown in Figure 2, and investigated their PL behavior and PL color-tuning potential.

## Results and Discussion

### Photoluminescence behavior of poly(methyl methacrylate) (PMMA) films

**Figure 3:**
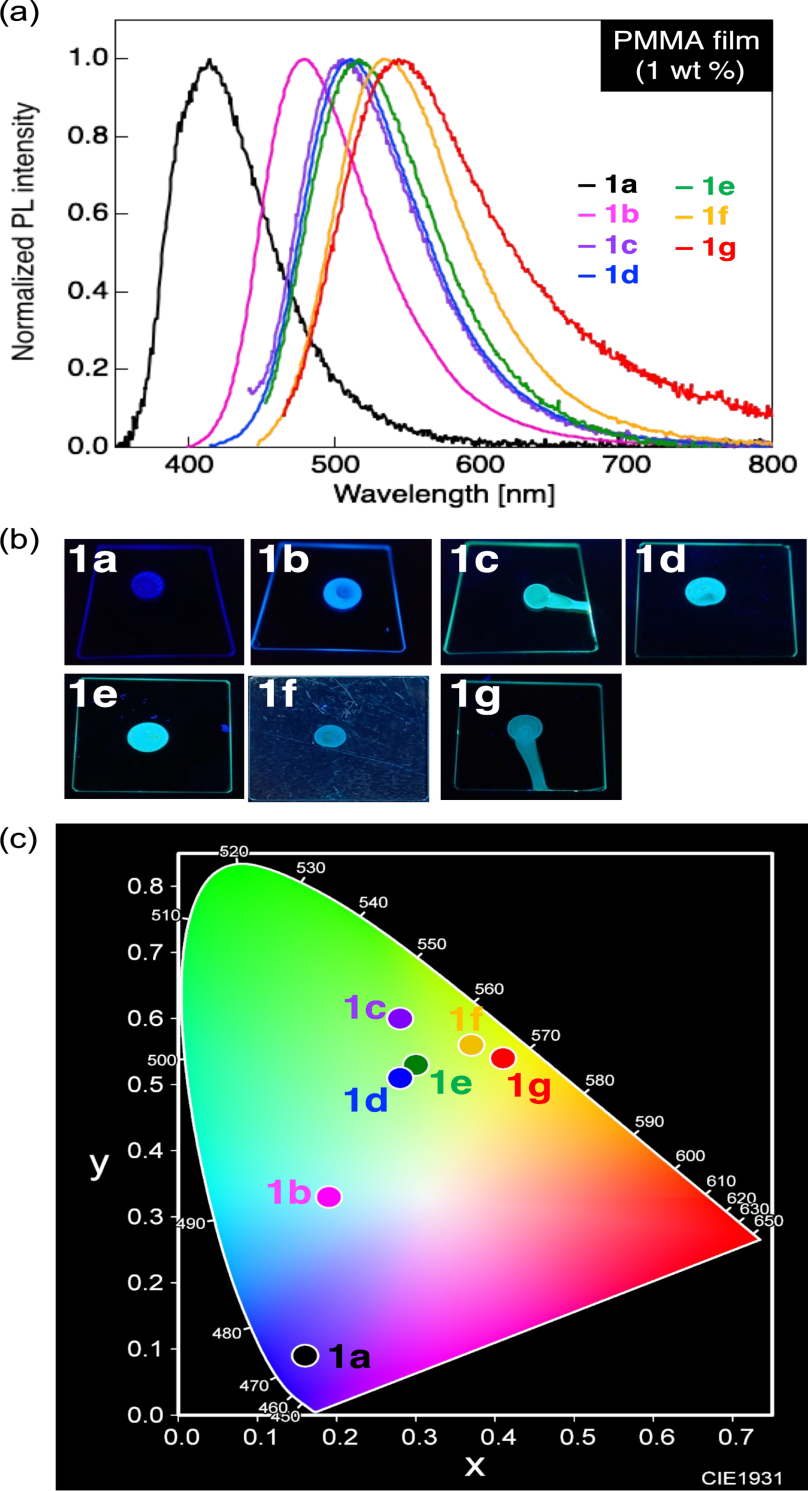
(a) PL spectra of the donor–π–acceptor (D–π–A)-type diphenylacetylene compounds **1a**–**g** contained in a poly(methyl methacrylate) (PMMA) dispersion film. (b) Photographs of the PMMA dispersion films under ultraviolet (UV) irradiation (λ_ex_ = 365 nm). (c) A PL color diagram defined by the Commission Internationale de l'Eclairage (CIE).

Initially, we tested the PL behavior of polymer dispersion films containing fluorinated diphenylacetylenes **1a**–**g** as a single component, as shown in Figure 3, because the PL behavior of fluorescent molecules dispersed in a polymer matrix generally differs from that in a dilute solution or the crystalline state. The PL wavelengths (λ_PL_), fluorescence quantum yields (Φ_PL_), fluorescence lifetimes (τ_PL_), and Commission Internationale de l'Eclairage (CIE) chromaticity coordinates of the PMMA dispersion films containing 1 wt % of compounds **1a**–**g** are summarized in Table 1.

**Table 1:** Photophysical data of compounds **1a**–**g** contained in a PMMA dispersion film.

	λ_ex_ [nm]	λ_PL_ [nm] (Φ_PL_)^a^	τ_PL_ [ns]	coordinate (*x*, *y*)^b^

**1a**	310	415 (0.76)	4.68	(0.16, 0.09)
**1b**	300	479 (1.00)	3.21	(0.19, 0.33)
**1c**	300	506 (0.84)	3.19	(0.28, 0.60)
**1d**	400	512 (0.66)	3.89	(0.28, 0.51)
**1e**	440	517 (0.54)	2.26	(0.30, 0.53)
**1f**	440	534 (0.66)	3.73	(0.37, 0.56)
**1g**	450	544 (0.03)	4.84	(0.41, 0.54)

^a^Measured using an integrating sphere; ^b^chromaticity coordinates defined by the CIE.

The PMMA dispersion films containing 1 wt % of compounds **1a**–**g** all exhibited a single PL band with λ_PL_s in the range of 415–544 nm, and their PL colors varied from dark blue to yellow with (*x, y*) coordinates of (0.16, 0.09) and (0.41, 0.54), respectively (Figure 3). A blueshift in the λ_PL_ ranging from 28 nm to a maximum of 126 nm was observed for all compounds contained in a PMMA film, based on a comparison with the λ_PL_ of the crystalline state shown in Figure 2. A decrease in the Φ_PL_ was observed for the PMMA dispersion films containing **1a** with a methoxy substituent, **1c** with a diphenylamino group, or **1g** with a phenothiazine unit. Judging from the fact that compounds **1a** and **1c** form a tight molecular packing via intermolecular H···F hydrogen bonds which suppress non-radiative deactivation in the crystalline state [20–21], we speculated that the polymer dispersion state had lost the intermolecular interactions, which accelerated the non-radiative deactivation process. On the other hand, the other derivatives, namely **1b** and **1d**–**f**, showed increased Φ_PL_ values in the PMMA films compared with those in the crystalline state, presumably due to a suppression of the formation of non-fluorescent twisted intramolecular charge transfer (TICT) states caused by the large ICT characteristics.

### Photoluminescence behavior of PMMA dispersion fluorescent films containing two fluorinated diphenylacetylenes

Based on the solid-state fluorescent molecule library **1a**–**g** developed by our group [20–23], we expected that white photoluminescent devices could be developed by precisely controlling the two-component mixture system of blue- and yellow-fluorescent molecules. From the perspective of both the PL color and Φ_PL_, we selected the methoxy-substituted compound **1a** as an effective blue-fluorescent molecule for use in a two-component mixing system. Among diphenylamino-substituted **1c** with chromaticity (*x, y*) coordinates of (0.28, 0.60), **1f** containing a tetramethyljulolidine unit with (*x, y*) coordinates of (0.37, 0.56), and **1g** containing a phenothiazine unit with (*x, y*) coordinates of (0.41, 0.54), **1c** and **1f** were finally selected as candidates for yellow-fluorescent molecules from the viewpoint of their Φ_PL_. Therefore, we investigated the PL behavior of PMMA dispersion films containing blue-fluorescent **1a**, green–yellow fluorescent **1c**, and yellow-fluorescent **1f**.

#### Photoluminescence behavior of PMMA dispersion films containing a mixture of blue fluorophore **1a** and green–yellow fluorophore **1c**

**Figure 4:**
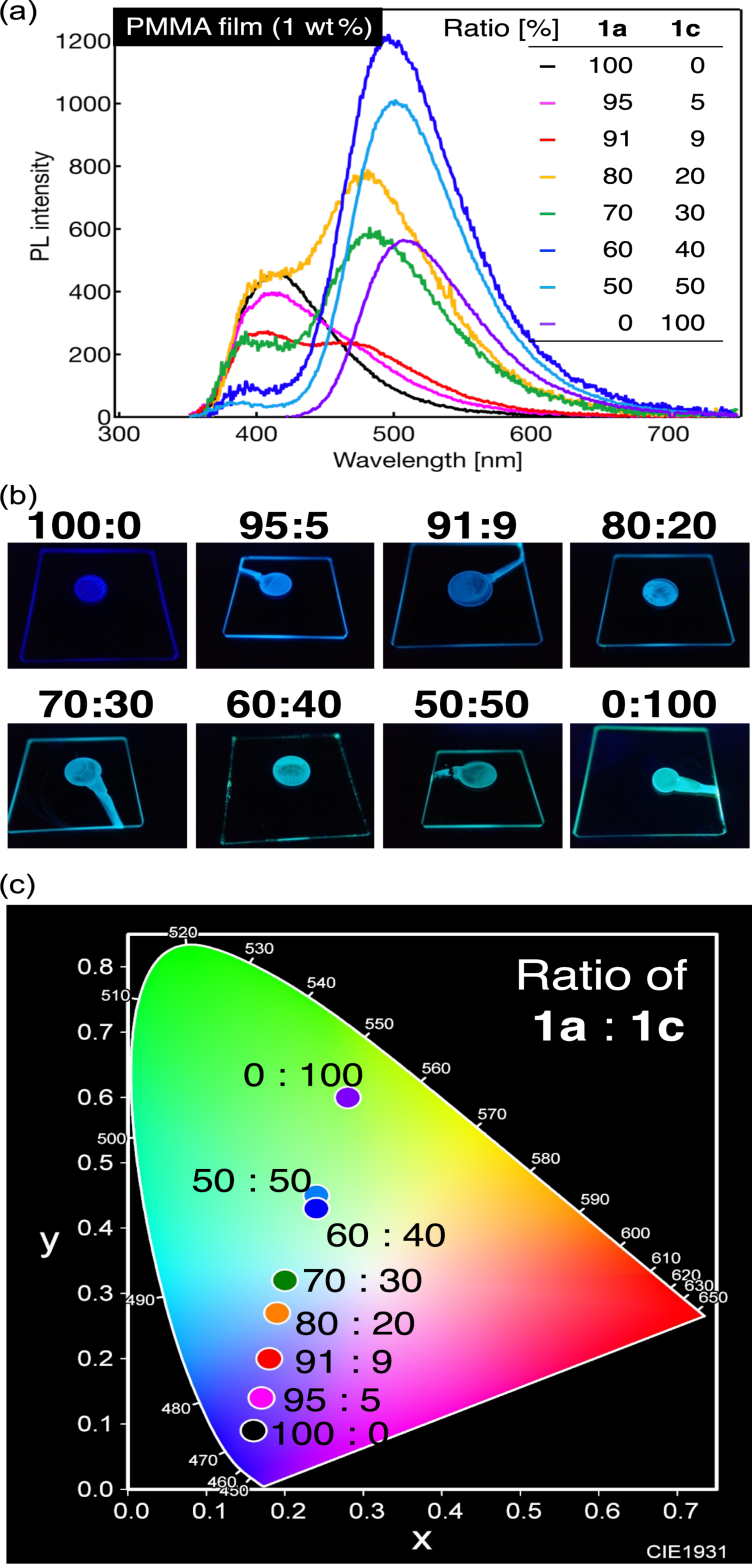
(a) PL spectra of PMMA dispersion films containing 1 wt % of blue fluorophore **1a** and green–yellow fluorophore **1c** in various weight ratios. (b) Photographs of the PMMA dispersion films under UV irradiation (λ_ex_ = 365 nm). (c) A CIE color diagram of the PL color of the PMMA dispersion films containing **1a** and **1c** in various ratios.

PMMA dispersion films containing 1 wt % of blue fluorophore **1a** and green–yellow fluorophore **1c** in various weight ratios were prepared. Their PL spectra and photophysical data are depicted in Figure 4 and Table 2 summarizes the collected photophysical data.

**Table 2:** Photophysical data of PMMA dispersion films containing 1 wt % of blue fluorophore **1a** and green–yellow fluorophore **1c** in various weight ratios.

Ratio of **1a**:**1c**	λ_PL_ [nm] (Φ_PL_)^a^	τ_PL_ [ns]	coordinate (*x*, *y*)^b^	*E*_FRET_ [%]^c^

100:0	415 (0.76)	4.68	(0.16, 0.09)	–
95:5	404 (0.97)	2.50	(0.17, 0.14)	47
91:9	404, 473 (0.92)	2.52	(0.18, 0.20)	46
80:20	400, 480 (0.93)	1.58	(0.19, 0.27)	66
70:30	392, 482 (0.76)	1.35	(0.20, 0.32)	71
60:40	496 (0.95)	2.73	(0.24, 0.43)	42
50:50	390, 504 (1.0)	2.79	(0.24, 0.45)	40
0:100	506 (0.84)	3.19	(0.28, 0.60)	–

^a^Measured using an integrating sphere; ^b^PL color coordinate defined by the CIE; ^c^fluorescence resonance energy transfer (FRET) efficiency (*E*_FRET_) = 1 − (τ_DA_/τ_D_).

As mentioned above, the PMMA film containing 1 wt % of fluorophore **1a** with a methoxy group exhibited a single PL band with a λ_PL_ at approximately 415 nm and dark blue PL with chromaticity coordinates of (0.16, 0.09). In contrast, the PMMA film containing 1 wt % of fluorophore **1c** with a diphenylamino group showed green–yellow PL with a λ_PL_ at approximately 506 nm and chromaticity coordinates of (0.28, 0.60). The PL behavior of the 1 wt % PMMA film blended with blue fluorophore **1a** and green–yellow fluorophore **1c** in a 50:50 ratio was evaluated, and two PL bands appeared, namely major and minor PL bands with λ_PL_s at approximately 504 and 390 nm, respectively. The fluorescent color of the PMMA film mixed in a 50:50 ratio showed light green–yellow PL with chromaticity coordinates of (0.24, 0.45), which suggests a rapid energy transfer of the excitation energy from blue fluorophore **1a** to green–yellow fluorophore **1c**. The absorption wavelengths and spectral shapes in the ultraviolet (UV)–visible absorption spectra of the PMMA films containing 1 wt % of **1a** and **1c** or **1f** as representative examples (Figure S2 in Supporting Information File 1) were similar to those in the corresponding excitation spectra, which clearly indicates that the PL originates from a single fluorophore. Using a 1 wt % PMMA film blended with **1a** and **1c** in an 80:20 ratio (Figure S2g in Supporting Information File 1), the excitation spectrum obtained by monitoring the long PL wavelength derived from **1c** was also in good agreement with the absorption spectrum of **1a** in the two-component film. This result also clearly suggests an energy transfer from **1a** to **1c**.

The fluorescence resonance energy transfer efficiency (*E*_FRET_) [30–31] was calculated from the ratio of the fluorescence lifetimes of the two- and high-energy-component films (Equation 1):


[1] $$E_{\text{FRET}}=\left(1-\frac{\tau_{\text{DA}}}{\tau_{\text{D}}}\right)\times 100,$$

where τ_DA_ and τ_D_ are the PL lifetimes of the two- and single-component films, respectively. An *E*_FRET_ of 40% for the two-component PMMA film blended in a 50:50 ratio indicates that the excitation energy was transferred relatively efficiently from **1a** to **1c**.

Next, the PL behavior of a PMMA film with a higher ratio of **1a** was investigated by mixing **1a**:**1c** in a ratio of 95:5. Contrary to the above result, a single PL band with a λ_PL_ at approximately 404 nm and a shoulder peak in the long-wavelength region were observed. The τ_PL_ of the PMMA film blended in a 95:5 ratio was 2.50 ns with an *E*_FRET_ of 47%, indicating that the PL band of **1c** was very small because of non-radiative deactivation, in addition to the low PL component of **1c**. The PL color of the PMMA film blended in a 95:5 ratio was dark blue, with color coordinates of (0.17, 0.14).

Based on these results, various mixing ratios of **1a** and **1c** ranging from 50:50 to 95:5 were investigated (91:9, 80:20, 70:30, and 60:40). Thus, a PMMA film mixed with **1a** and **1c** in a ratio of 91:9 exhibited two PL bands with λ_PL_s at approximately 404 and 473 nm, respectively. The PL bands on the short- and long-wavelength sides were considered to be derived from the emissions of **1a** and **1c**, respectively. For the PL of the PMMA film blended in a 91:9 ratio, the τ_PL_ and *E*_FRET_ values were 2.52 ns and 46%, respectively. Although the PMMA film blended in a 91:9 ratio was observed to have two distinct PL bands, the PL color turned blue with coordinates of (0.18, 0.20). Furthermore, when the mixing ratio of **1a** to **1c** was changed to 80:20, 70:30, and 60:40, the relative intensity of the long-wavelength PL band originating from the PL of **1c** relative to that of **1a** increased with the increasing mixing ratio of **1c**. The τ_PL_ values were in the range of 1.35–2.73 ns in these blends and the *E*_FRET_ values were in the range of 42–71%. These results indicate a good energy transfer for all **1a** and **1c** mixtures. Based on the PL spectra of the PMMA films blended in each mixing ratio, the PL colors of the 80:20 and 70:30 blends were light blue with coordinates of (0.19, 0.27) and (0.20, 0.32), respectively, and the PL color of the 60:40 blend was green–blue with coordinates of (0.24, 0.43). The PL color of the PMMA films containing the **1a**/**1c** mixture prepared in each mixing ratio varied along a straight line connecting the color coordinates of the PMMA films containing the individual component **1a** or **1c**. In other words, tuning the PL color was possible by controlling the mixing ratio. In addition, when the mixing ratio of **1a** to **1c** was 70:30, the chromaticity coordinates were (0.20, 0.32), which are relatively close to the ideal color coordinates of (0.33, 0.33) for white. The results for the PMMA film containing a mixture of **1a** and **1c** indicate that the use of fluorophores with PL bands on the longer-wavelength side is more effective in approaching the ideal white color than green–yellow fluorophore **1c**.

#### Photoluminescence behavior of PMMA dispersion films containing a mixture of blue fluorophore **1a** and yellow fluorophore **1f**

**Figure 5:**
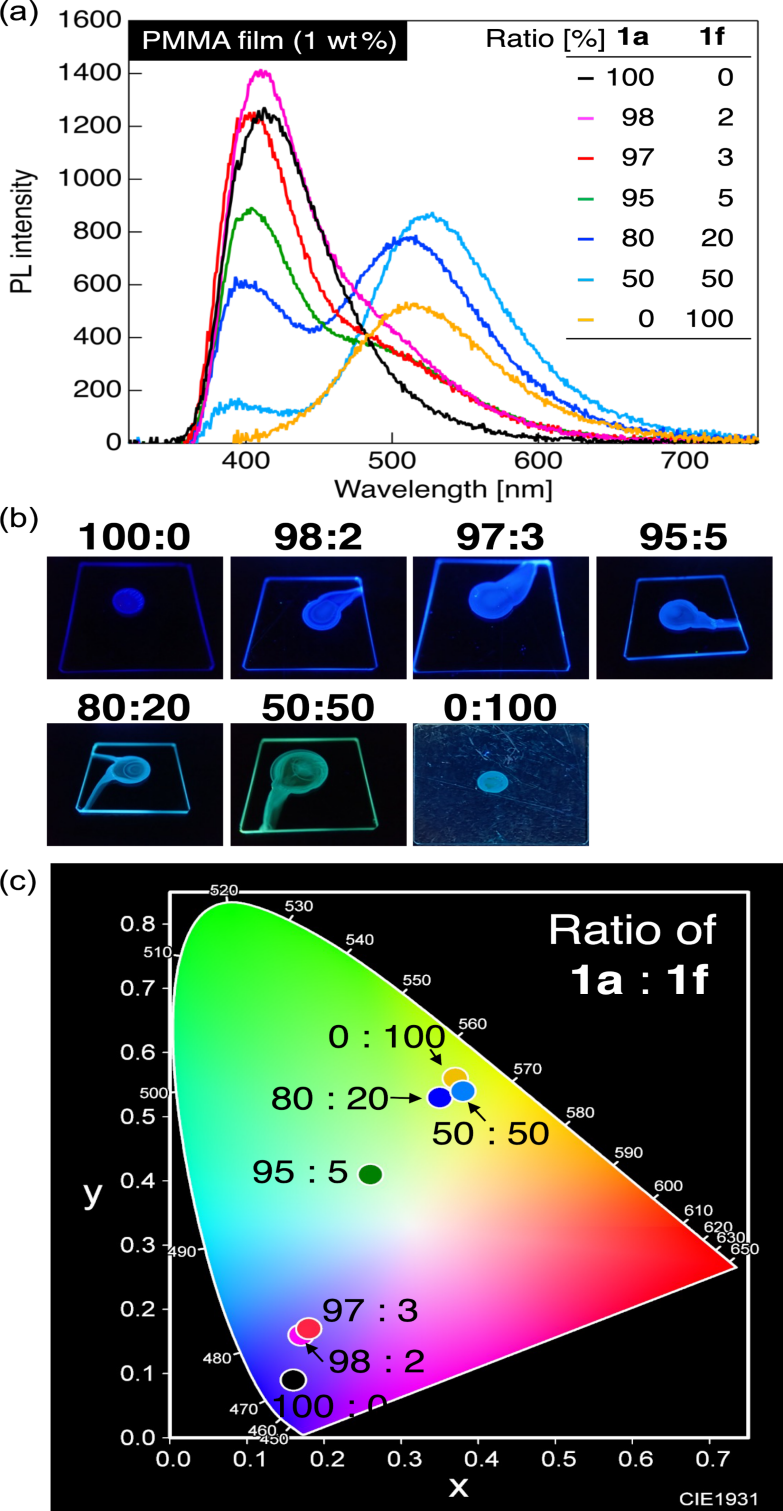
(a) PL spectra of PMMA dispersion films containing 1 wt % of blue fluorophore **1a** and yellow fluorophore **1f** in various weight ratios. (b) Photographs of their PMMA dispersion films under UV irradiation (λ_ex_ = 365 nm). (c) A CIE color diagram of the PL color of PMMA dispersion films containing **1a** and **1f** in various weight ratios.

Based on our investigation of the PL behavior of PMMA dispersion films containing a mixture of **1a** and **1c**, we investigated the PL properties of PMMA dispersion films containing a mixture of blue fluorophore **1a** and yellow fluorophore **1f** (Figure 5). The collected photophysical data are summarized in Table 3.

**Table 3:** Photophysical data of PMMA dispersion films containing 1 wt % of blue fluorophore **1a** and yellow fluorophore **1f** in various weight ratios.

Ratio of **1a**:**1f**	λ_PL_ [nm] (Φ_PL_)^a^	τ_PL_ [ns]	coordinate (*x*, *y*)^b^	*E*_FRET_ [%]^c^

100:0	415 (0.76)	4.68	(0.16, 0.09)	–
98:2	407 (0.76)	1.96	(0.17, 0.16)	58
97:3	417 (0.82)	2.04	(0.18, 0.17)	56
95:5	401, 520 (0.94)	2.48	(0.26, 0.41)	47
80:20	384, 530 (1.0)	2.53	(0.35, 0.53)	46
50:50	392, 541 (1.0)	2.12	(0.38, 0.54)	55
0:100	534 (0.66)	3.73	(0.37, 0.56)	–

^a^Measured using an integrating sphere; ^b^PL color coordinates defined by the CIE; ^c^*E*_FRET_ = 1 – (τ_DA_/τ_D_).

Unlike the PMMA film containing green–yellow fluorophore **1c**, the 1 wt % PMMA film containing **1f** with a tetramethyljulolidine backbone exhibited yellow PL with a λ_PL_ at approximately 534 nm and chromaticity coordinates of (0.37, 0.56). When blue fluorophore **1a** and yellow fluorophore **1f** were blended in ratios of 50:50 and 80:20, the 1 wt % PMMA films containing this mixture showed a major PL band derived from **1f** with a λ_PL_ at 530–541 nm, along with a minor PL band derived from **1a** at 384–392 nm. The fluorescent color of the PMMA film containing this blend was yellow with chromaticity coordinates of (0.38, 0.54) for the blend in a 50:50 ratio, and green–yellow with chromaticity coordinates of (0.35, 0.53) for the blend in an 80:20 ratio. The τ_PL_ values of the PMMA films containing blends with ratios of 50:50 and 80:20 were 2.12 and 2.53 ns, respectively, and their *E*_FRET_ values were 55% and 46%. Both mixtures showed a relatively fast energy transfer, which likely caused the PL of **1f** to be the major component. Furthermore, the excitation spectrum obtained by monitoring the long PL wavelength originating from **1f** using a 1 wt % PMMA film blended with **1a** and **1f** in an 80:20 ratio was also consistent with the corresponding absorption spectrum of **1a** (Figure S2h in Supporting Information File 1). This result clearly indicates an energy transfer from **1a** to **1f**. Next, we evaluated the PL properties of the PMMA film blended in a 95:5 ratio, which had a higher weight ratio of **1a**. As a result, two distinct PL bands with λ_PL_s at approximately 401 and 520 nm appeared, which correspond to the PL derived from blue-fluorescent **1a** and yellow-fluorescent **1f**, respectively. The τ_PL_ was measured to be 2.48 ns, indicating an increase in the short-lived **1a** component. The *E*_FRET_ was also calculated to be 47%, which indicates that energy transfer occurred smoothly from **1a** to **1f**. The PL color of the PMMA film blended in a 95:5 ratio was pale green–yellow with chromaticity coordinates of (0.26, 0.41).

Further studies were conducted on PMMA films with increased content of **1a** and blends of **1a** and **1f** in ratios of 97:3 and 98:2. Compared with the PL spectrum of the PMMA film blended in a 95:5 ratio, the PL intensity of the short-wavelength PL band derived from the emission of **1a** increased with increasing contents of **1a**. The τ_PL_ values of the PMMA films blended in ratios of 97:3 and 98:2 were 2.04 and 1.96 ns, respectively, and their *E*_FRET_ values were 56% and 58%. These results indicate that a relatively efficient transfer of energy occurred from **1a** to **1f**. However, the PL color of the PMMA films containing the blend was light purple with chromaticity coordinates of (0.18, 0.17) for the 97:3 ratio, and blue with chromaticity coordinates of (0.17, 0.16) for the 98:2 ratio. Similar to the PL color trend in the PMMA films blended with **1a** and **1c**, the PL colors of the PMMA films blended with **1a** and **1f** were located on a line connecting them, suggesting that tuning the PL color from blue to yellow is possible by precisely adjusting the mixing ratios of **1a** and **1f**. When the mixing ratio of **1a** to **1f** was 95:5, the chromaticity coordinates were (0.26, 0.41). The PL of the PMMA films blended with the **1a**/**1f** binary mixture was relatively close to white, although it was slightly different from the ideal white point.

## Conclusion

In conclusion, we prepared PMMA dispersion films with a single component of fluorinated diphenylacetylene or a blend of two fluorinated diphenylacetylenes at a concentration of 1 wt % and investigated their PL behavior in detail. Among the fluorinated diphenylacetylene libraries with excellent solid-state luminescent properties, the PMMA dispersion film containing a methoxy-substituted fluorinated diphenylacetylene exhibited dark blue PL, the PMMA film containing a diphenylamino-substituted fluorinated diphenylacetylene exhibited green–yellow PL, and the PMMA film containing a fluorinated diphenylacetylene with a tetramethyljulolidine skeleton exhibited yellow PL. Intensive investigation of the PL behavior of these PMMA films blended as a binary mixture of blue and green–yellow or yellow fluorophores showed smooth energy transfer from the high-energy fluorescent component, that is, the methoxy-substituted fluorinated diphenylacetylene, to the yellow–green or yellow fluorophore, respectively. The PL behavior of each PMMA film blended as a binary mixture was investigated by varying the mixing ratio, and a wide range of PL colors, including dark blue, green–yellow, and yellow with chromaticity coordinates of (0.16, 0.09), (0.28, 0.60), and (0.37, 0.56), respectively, were obtained. In addition, both PMMA films containing the binary blends had color coordinates of (0.20, 0.32) and (0.26, 0.41) by precisely controlling the mixing ratio, and an approach to the white point (0.33, 0.33) of pure white emission could be observed. Further fine-tuning of the mixing ratios and PMMA films containing red–green–blue ternary mixtures holds promise for developing white-light-emitting materials with higher color purities and more diverse PL color tunings.

## Experimental

### Fabrication of PMMA dispersion films

PMMA films containing one of two fluorinated diphenylacetylenes were prepared using an Opticoat MS-B100 spin coater (MIKASA, Japan). The mother liquor for the thin-film deposition was prepared as follows: it was mounted on a thoroughly cleaned glass slide and clamped to the upper rotating plate of the spin coater. PMMA (100 mg) and the fluorophore (1.0 wt %) were dissolved in CHCl_3_ (3 mL), and a few drops of the solution were dropped onto a glass substrate. The top plate of the spin coater was rotated sequentially at 500 rpm for 5 s, 750 rpm for 5 s, and 1200 rpm for 10 s. For photophysical measurements, thin and smooth films were prepared by spin-coating followed by solvent evaporation.

### Photophysical properties

The PL spectra and quantum yields were measured using a Quantaurus-QY C11347-01 absolute PL quantum yield spectrometer (Hamamatsu Photonics, Japan). The PL lifetime was measured using a Quantaurus-Tau C11367-34 fluorescence lifetime spectrometer (Hamamatsu Photonics, Japan).

## Supporting Information

File 1 PL spectra and PL decay profiles of PMMA films containing 1 wt % of compounds or binary mixtures.

## Data Availability

All data that supports the findings of this study is available in the published article and/or the supporting information of this article.
