# Supplementary material for: Photoluminescence color-tuning with polymer-dispersed fluorescent films containing two fluorinated diphenylacetylene-type fluorophores
Source: Beilstein J Org Chem. 2024 Oct 23;20:2682–90. doi: 10.3762/bjoc.20.225 (PMC11514438; doi:10.3762/bjoc.20.225)
Supplement: File 1 — PL spectra and PL decay profiles of PMMA films containing 1 wt % of compounds or binary mixtures. [file Beilstein_J_Org_Chem-20-2682-s001.pdf]

BEILSTEIN JOURNAL OF ORGANIC CHEMISTRY

Supporting Information

for

Photoluminescence color-tuning with polymer-dispersed fluorescent films containing two fluorinated diphenylacetylene-type fluorophores

Kazuki Kobayashi, Shigeyuki Yamada, Motohiro Yasui and Tsutomu Konno

Beilstein J. Org. Chem. **2024,** *20,* 2682–2690. doi:10.3762/bjoc.20.225

PL spectra and PL decay profiles of PMMA films containing 1 wt % of compounds or binary mixtures

License and Terms: This is a supporting information file under the terms of the Creative Commons Attribution License (https://creativecommons.org/licenses/by/4.0). Please note that the reuse, redistribution and reproduction in particular requires that the author(s) and source are credited and that individual graphics may be subject to special legal provisions.
The license is subject to the *Beilstein Journal of Organic Chemistry* terms and conditions: (https://www.beilstein-journals.org/bjoc/terms)

Table of contents

Photophysical characteristic of **1a**–**g** in PMMA film S2

Absorption and excitation spectra of **1a**, **1c**, and **1f** in PMMA film S3

PL decay profile of **1a**–**g** in PMMA film S5

Photophysical characteristic of **1a**/**1c** binary mixtures in PMMA film S6

PL decay profile of **1a**/**1c** binary mixtures in PMMA film S7

Photophysical characteristic of **1a**/**1f** binary mixtures in PMMA film............................S8

PL decay profile of **1a**/**1f** binary mixtures in PMMA film..S9

References S9

Photophysical data for the fluorinated diphenylacetylene derivatives **1a**–**g** used in this study have been already published [1-4].

Photophysical characteristics in PMMA films

Thin films of synthetic derivatives were prepared using an Opticoat MS-B100 spin coater (MIKASA, Japan). The solutions of PMMA (100 mg) in $CHCl_3$ (3 mL) containing 1.0 wt % per weight with varying ratios of derivatives were prepared. Then, the thoroughly cleaned glass slide was mounted and clamped on the top rotating plate of a spin coater. Few drops of a solution of PMMA in $CHCl_3$ containing the calculated amounts of derivatives were placed on the glass slide and the top plate of spin coater kept rotating at the speed of 500 revolutions per minute (rpm) for 5 seconds, then at 750 rpm for an additional 5 seconds, and finally at 1200 rpm for 10 seconds. The solvent evaporated and a thin smooth film was seen deposited on the glass slide. The prepared films were used for photophysical measurements without further drying the solvent.

UV–vis absorption spectra were recorded using a JASCO V-530 and V-750 absorption spectrometer (JASCO, Japan). PL spectra of the solution was measured using an Rf-6000 (Shimadzu, Japan). Photoluminescence quantum yields were measured using Quantaurus-QY C11347-01 series (Hamamatsu Photonics, Japan). PL lifetime was measured using a Quantaurus-Tau fluorescence lifetime spectrometer C11367-34 (Hamamatsu Photonics, Japan).

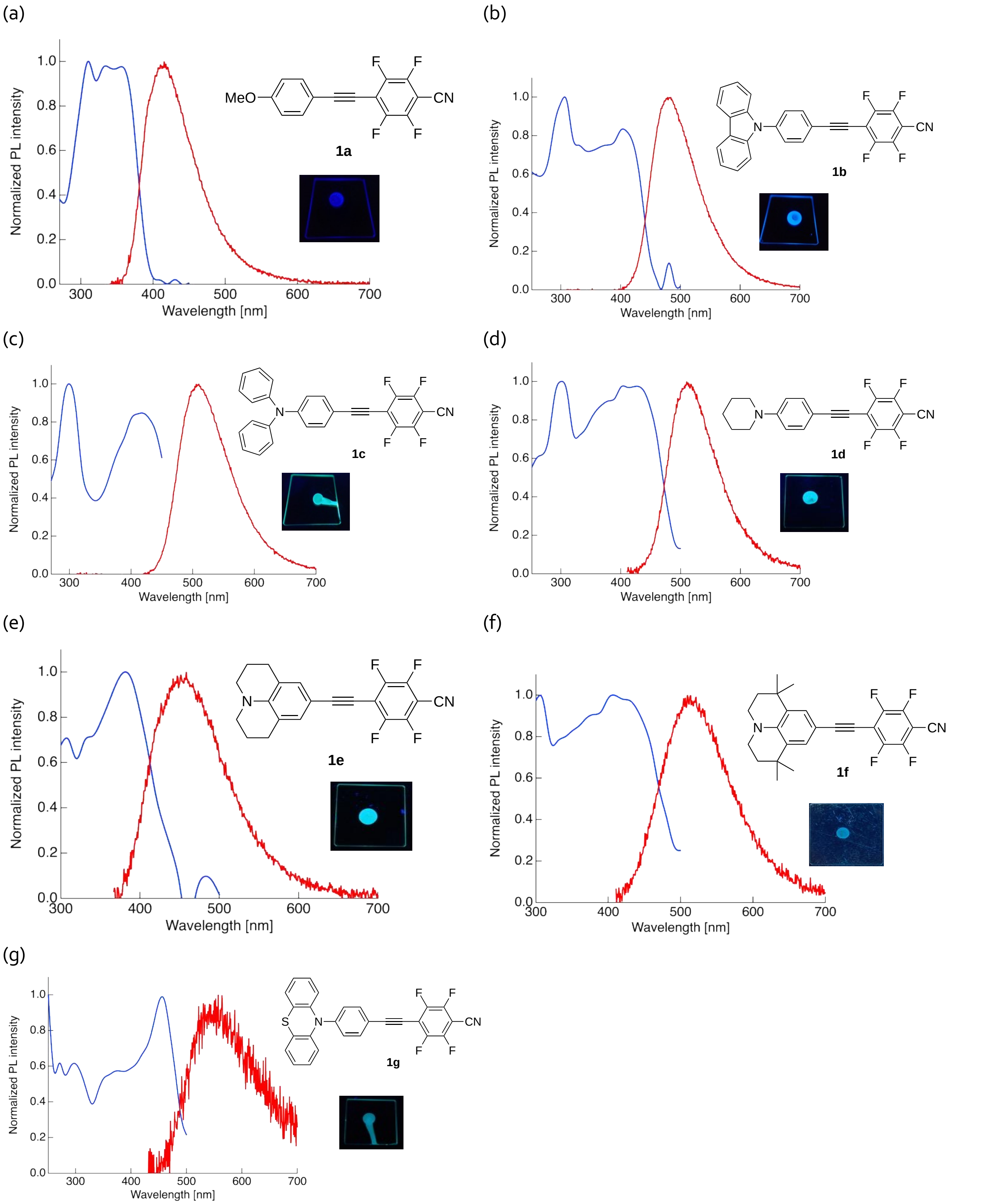

Figure S1: Excitation and PL spectra of PMMA film containing 1 wt % of (a) **1a** (λ_{em} = 415 nm; λ_{ex} = 310 nm), (b) **1b** (λ_{em} = 479 nm; λ_{ex} = 300 nm), (c) **1c** (λ_{em} = 506 nm; λ_{ex} = 410 nm), (d) **1d** (λ_{em} = 512 nm; λ_{ex} = 400 nm), (e) **1e** (λ_{em} = 517 nm; λ_{ex} = 390 nm), (f) **1f**, (λ_{em} = 534 nm; λ_{ex} = 410 nm) and (g) **1g** (λ_{em} = 544 nm; λ_{ex} = 450 nm). Photographs of PL behavior under UV irradiation (λ_{ex} = 365 nm).

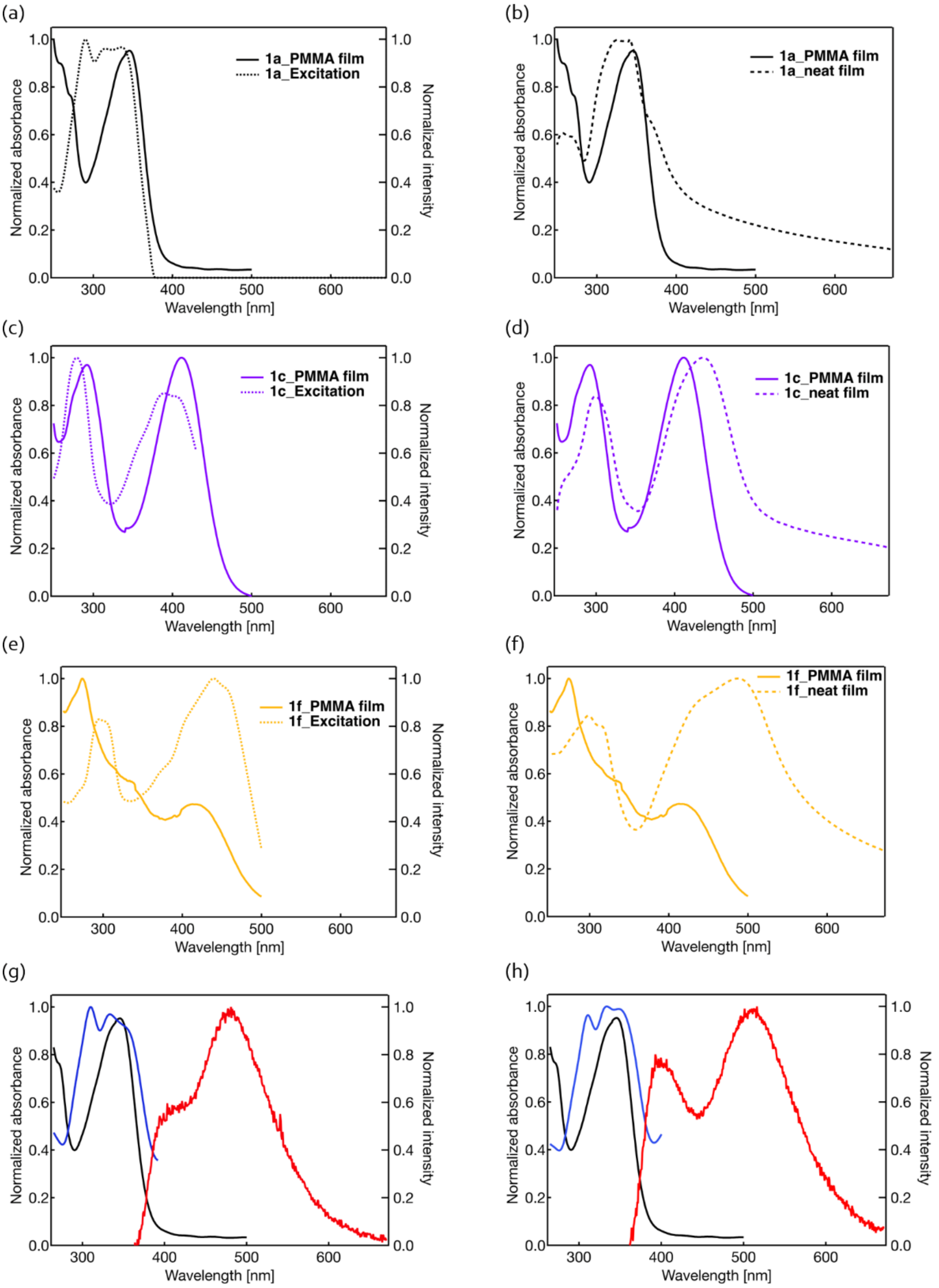

Figure S2: UV–vis absorption and excitation spectra of PMMA film containing 1 wt % of (a) **1a** (c) **1c** (e) **1f**. UV–vis absorption of PMMA film and neat film of (b) **1a**, (d) **1c**, (f) **1f**. All excitation spectra were measured with fluorescence maxima monitored. UV–vis absorption (black), excitation (blue) and PL spectra (red) of PMMA film containing 1 wt % of **1a** and **1c** blends in (g) 80:20 ratio (λ_{em} = 480 nm), and 1**a** and **1f** blends in (h) 80:20 ratio (λ_{em} = 530 nm).

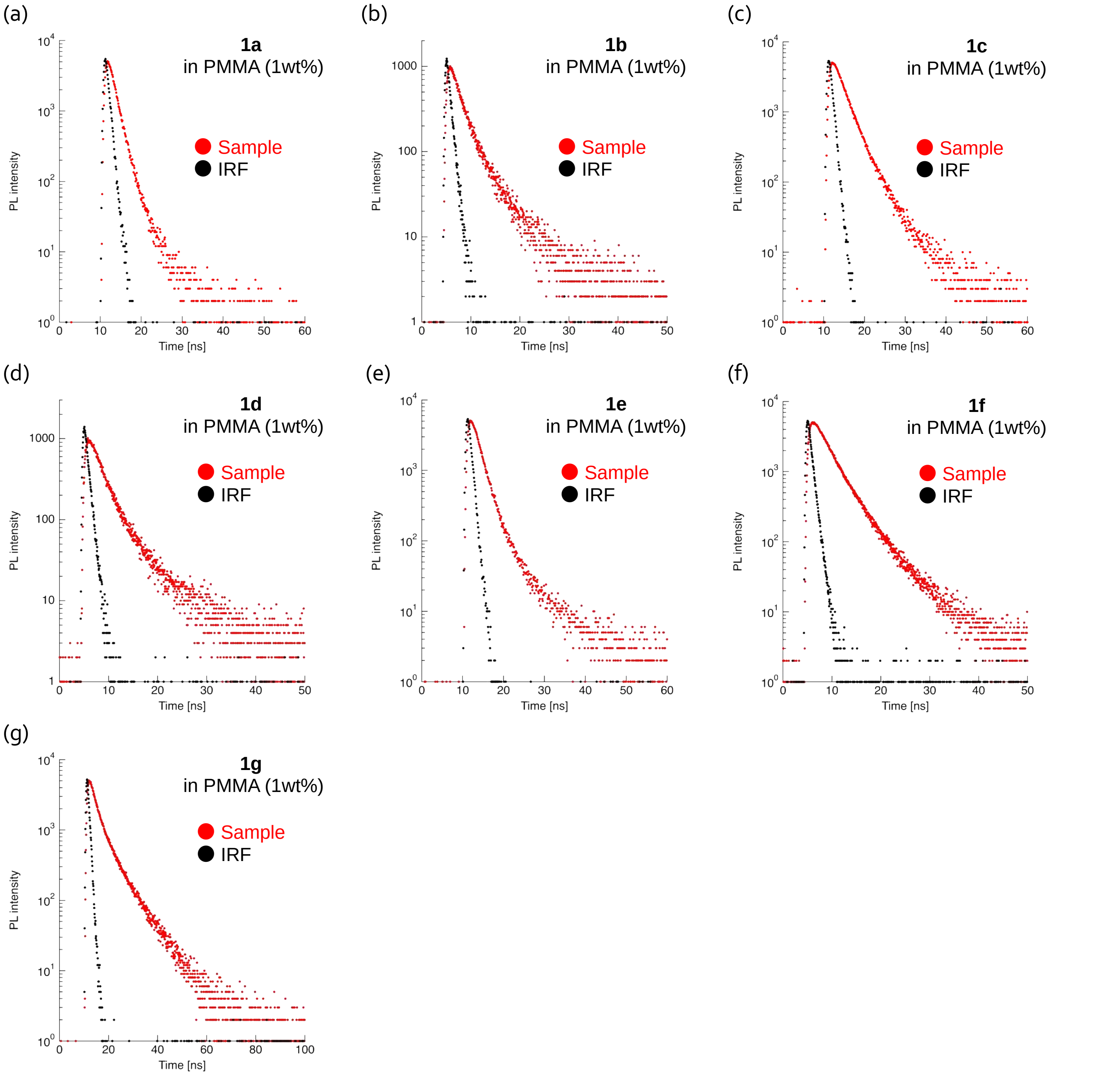

Figure S3: PL decay profiles of PMMA film containing 1 wt % of (a) **1a** (λ_{ex} = 340 nm), (b) **1b** (λ_{ex} = 280 nm), (c) **1c** (λ_{ex} = 340 nm), (d) **1d** (λ_{ex} = 340 nm), (e) **1e** (λ_{ex} = 340 nm), (f) **1f**, (λ_{ex} = 340 nm) and (g) **1g** (λ_{ex} = 340 nm). Monitored by PL maximum wavelength.

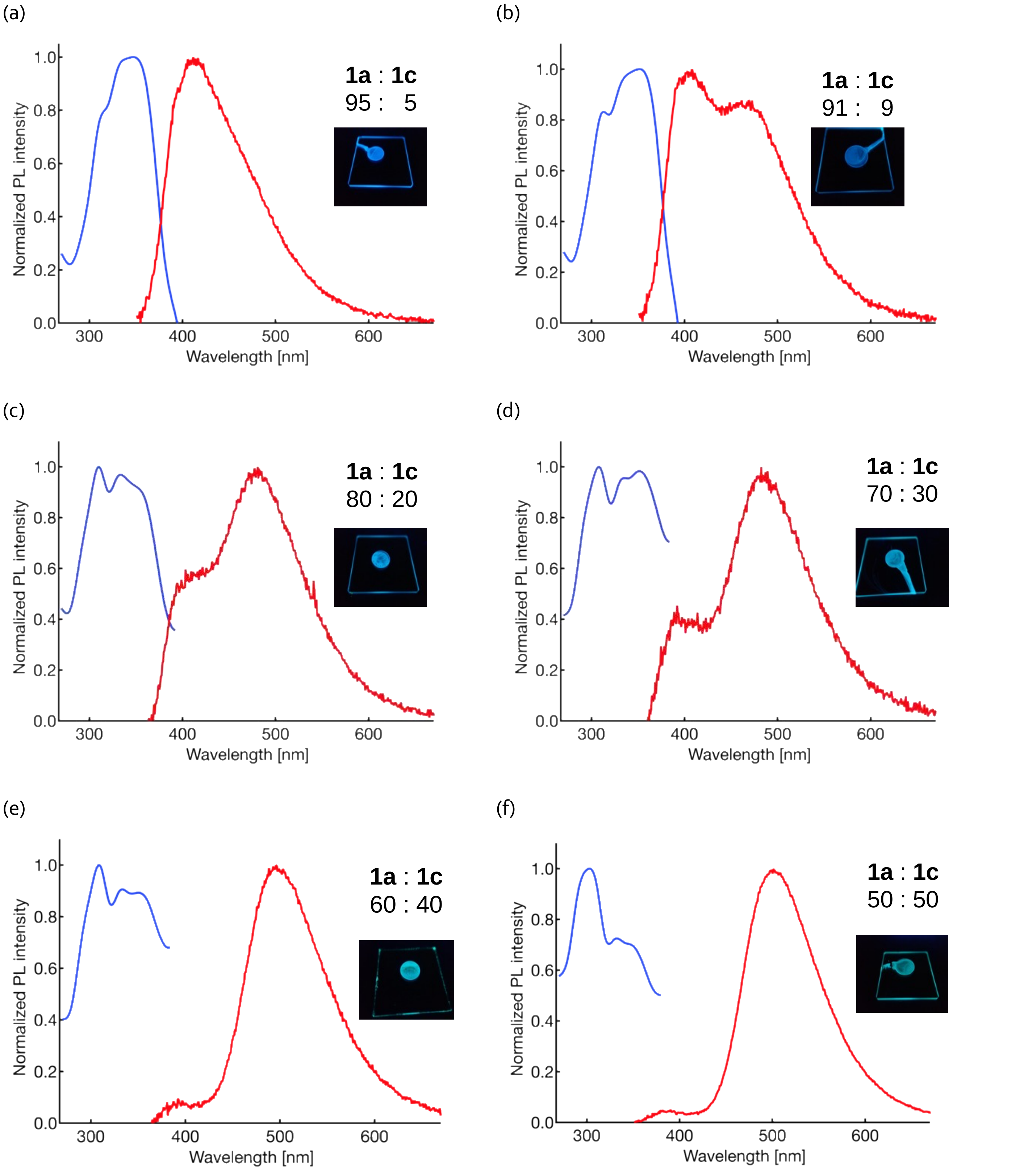

Figure S4: Excitation and PL spectra (λ_{ex} = 310 nm) of PMMA film containing 1 wt % of **1a** and **1c** blends in (a) 95:5 (λ_{em} = 404 nm), (b) 91:9 (λ_{em} = 404 nm), (c) 80:20 (λ_{em} = 480 nm), (d) 70:30 (λ_{em} = 482 nm), (e) 60:40 (λ_{em} = 496 nm), and (f) 50:50 ratio (λ_{em} = 504 nm). Photographs of PL behavior under UV irradiation (λ_{ex} = 365 nm).

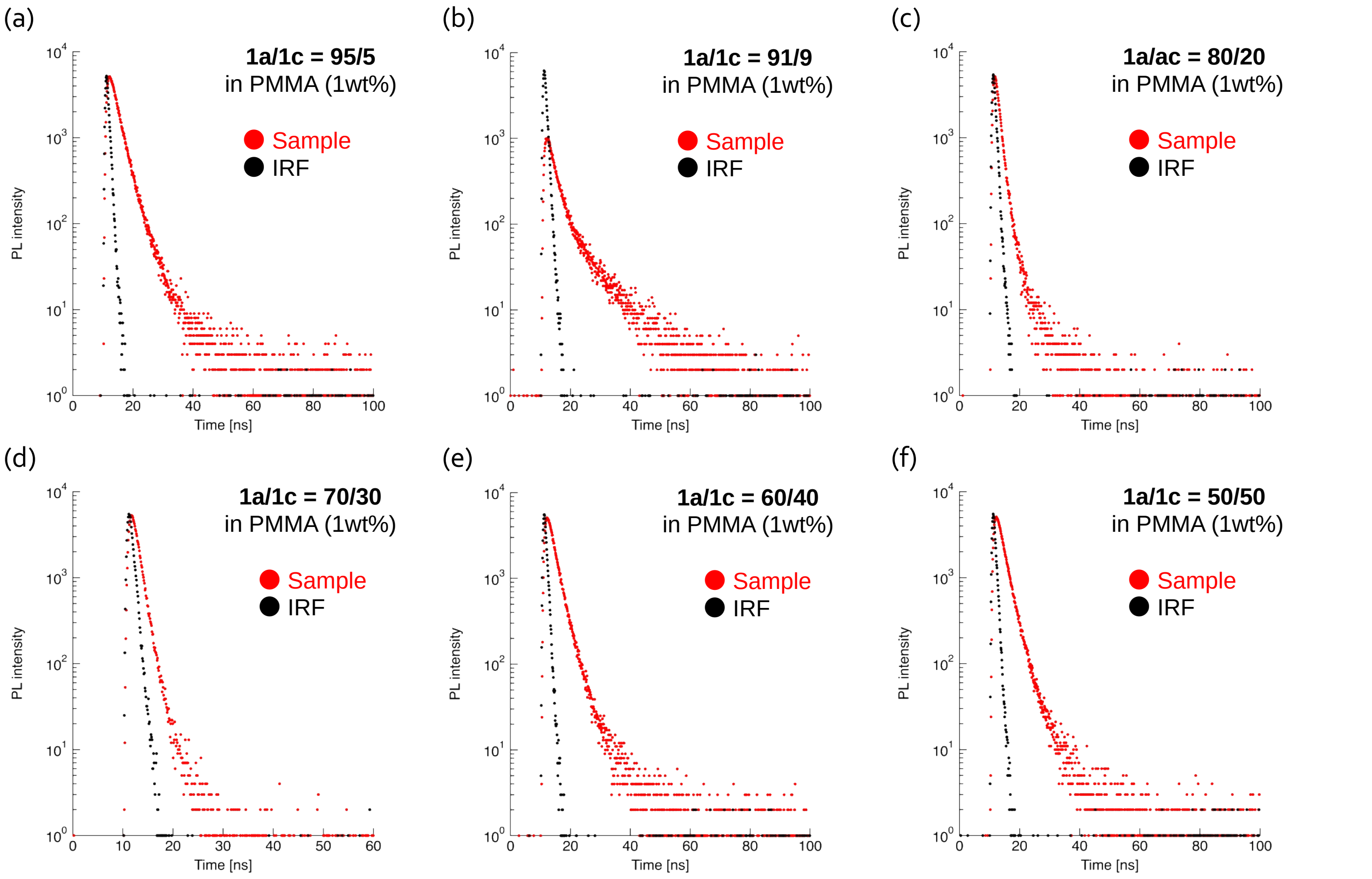

Figure S5: PL decay profiles of PMMA film containing 1 wt % of **1a** and **1c** blends in (a) 95:5, (b) 91:9, (c) 80:20, (d) 70:30, (e) 60:40, and (f) 50:50 ratio. All samples were excited by 340 nm. Monitored by fluorescence maximum wavelength.

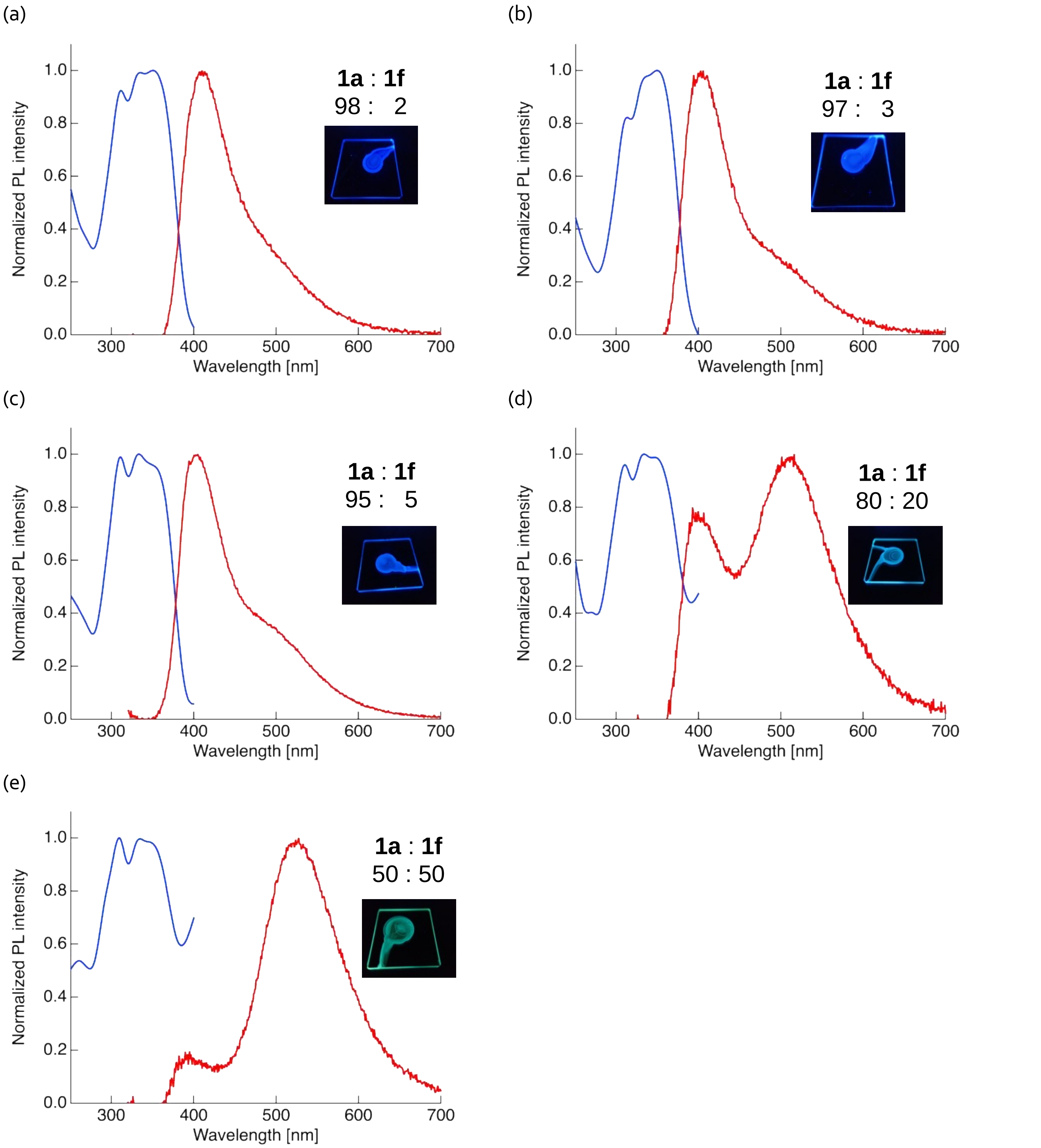

Figure S6: Excitation and PL spectra (λ_{ex} = 310 nm) of PMMA film containing 1 wt % of **1a** and **1f** blends in (a) 98:2 (λ_{em} = 407 nm), (b) 97:3 (λ_{em} = 417 nm), (c) 95:5 (λ_{em} = 401 nm), (d) 80:20 (λ_{em} = 530 nm), and (f) 50:50 ratio (λ_{em} = 541 nm). Photographs of PL behavior under UV irradiation (λ_{ex} = 365 nm).

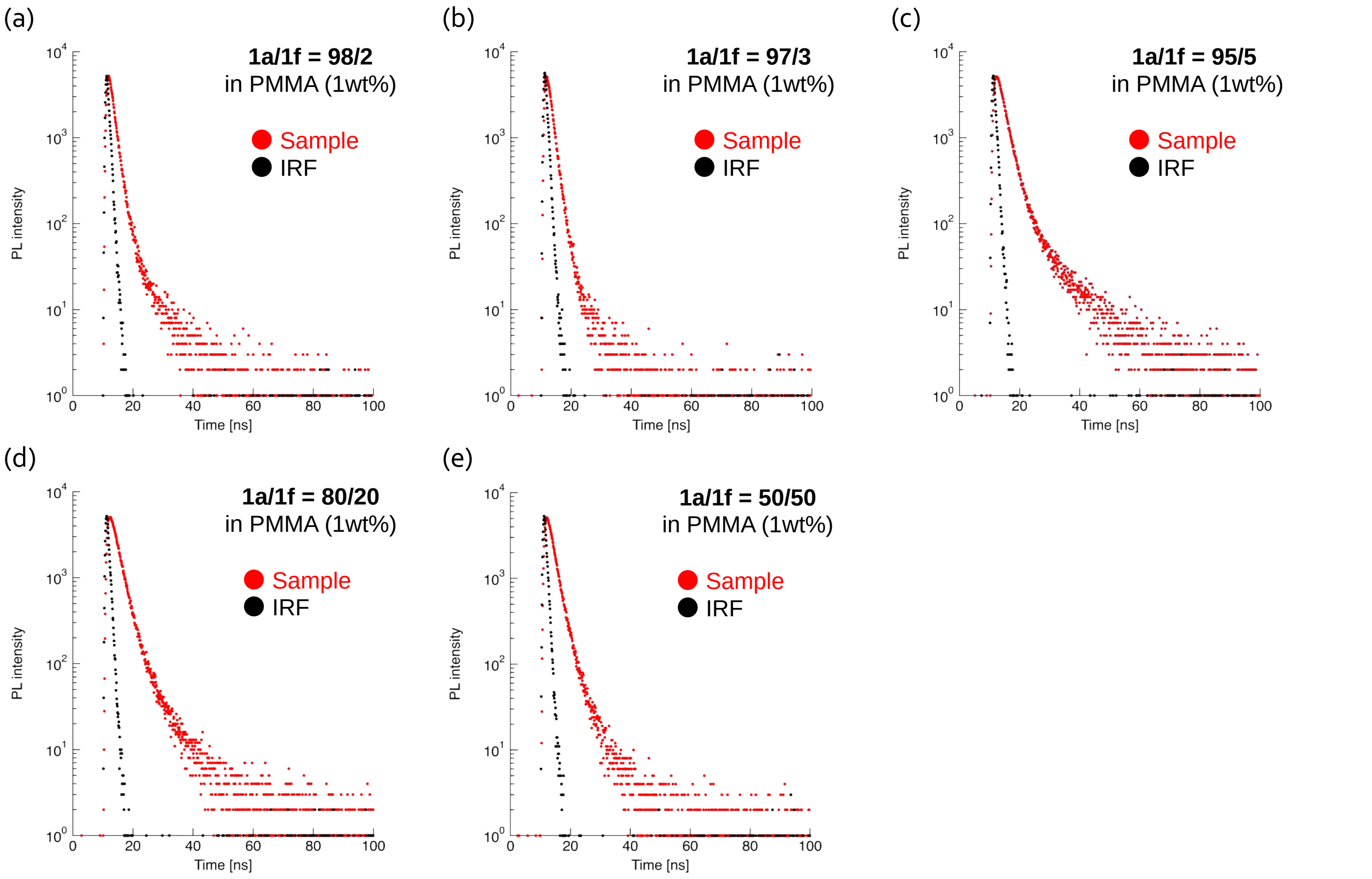

Figure S7: PL decay profiles of PMMA film containing 1 wt % of **1a** and **1f** blends in (a) 98:2, (b) 97:3, (c) 95:5, (d) 80:20, and (f) 50:50 ratio. Photographs of PL behavior under UV irradiation (λ_{ex} = 365 nm). All samples were excited by 340 nm. Monitored by fluorescence maximum wavelength.

References:

1. Morita, M.; Yamada, S.; Konno, T. *New J. Chem.* **2020,** *44,* 6704–6708. doi:10.1039/D0NJ01268H
2. Yamada, S.; Kobayashi, K.; Morita, M.; Konno, T. *CrystEngComm* **2022,** *24,* 936–941. doi:10.1039/D1CE01671G
3. Yamada, S.; Kobayashi, K.; Konno, T. *Molecules* **2022,** *27,* 5782. doi:10.3390/molecules27185782
4. Kobayashi, K.; Yamada, S.; Sakurai, T.; Yasui, M.; Konno, T. *J. Mater. Chem. C*, submitted.
